# Integrating traditional omics and AI-driven approaches for discovery and validation of novel MicroRNA biomarkers and therapeutic targets in thyroid cancer

**DOI:** 10.3389/fphar.2025.1727032

**Published:** 2026-01-28

**Authors:** Yi Wan, Dan Xie, Min Zhang, Shiyu Yang, Zhantian Zhang, Xiaomin Fu, Meiling Wang, Yongfu Zhao

**Affiliations:** 1 Department of Thyroid Surgery, The Second Hospital of Dalian Medical University, Dalian, Liaoning, China; 2 Department of Breast and Thyroid surgery, Affiliated Zhongshan Hospital of Dalian University, Dalian, Liaoning, China; 3 Department of Anesthesiology,Central Hospital of Dalian University of Technology (Dalian Municipal Central Hospital), Dalian, Liaoning, China

**Keywords:** machine learning, biomarker discovery, drug target validation, MicroRNA therapeutics, single-cell RNA sequencing, thyroid cancer, omics integration, therapeutic mechanisms

## Abstract

**Background:**

The discovery of reliable biomarkers and therapeutic targets remains a critical challenge in thyroid cancer management. This study demonstrates the value of integrating traditional omics technologies with artificial intelligence approaches and single-cell validation to identify novel microRNA-based biomarkers and drug targets. We hypothesized that combining meta-analysis of bulk transcriptomics, machine learning-driven feature selection, and single-cell spatial mapping would enhance biomarker discovery and validation compared to using either approach independently.

**Methods:**

We employed a hybrid strategy integrating traditional transcriptomic analysis with AI-driven methods. Meta-analysis of three bulk RNA-seq datasets (GSE65144, GSE33630, GSE50901) was performed using effect size analysis, followed by machine learning-based forward feature selection to identify optimal biomarker combinations. Single-cell RNA-seq data (GSE184362, 196,145 cells from 23 thyroid cancer samples) provided cell-type-specific validation and immune microenvironment profiling. Comprehensive experimental validation was conducted using TPC-1 and BHT101 cell lines through miR-6756-5p overexpression and CRISPRi-mediated knockdown, including functional assays and xenograft experiments to establish therapeutic potential.

**Results:**

The AI-enhanced meta-analysis identified a four-gene diagnostic panel (BID, MIR6756, ITM2A, TGM2) achieving exceptional performance with AUC values of 1.0 and 0.99 in training sets and 0.74 in independent validation. Single-cell analysis of 50,000 cells revealed six major cell types with significant immune infiltration (61.9%), providing crucial cell-type specificity for the identified biomarkers. BID and ITM2A showed predominantly epithelial expression, while TGM2 was enriched in immune and stromal compartments, demonstrating multi-cellular biomarker patterns. Immune microenvironment analysis revealed distinct CD8+/CD4+ T cell ratios between metastatic and non-metastatic samples. hsa-miR-6756-5p, identified through this integrated approach, exhibited tumor-specific expression and demonstrated oncogenic properties by promoting proliferation, colony formation, migration, and invasion *in vitro*, while enhancing tumor growth *in vivo*, validating it as a novel therapeutic target.

**Discussion:**

Our study exemplifies the synergistic value of integrating traditional omics approaches with AI-driven analytics for biomarker and drug target discovery. The combination of machine learning-based feature selection from bulk transcriptomics with single-cell spatial validation addresses limitations of each approach used independently. This integrated framework successfully identified has-miR-6756-5p as both a diagnostic biomarker and therapeutic target, demonstrating how traditional experimental validation coupled with computational prediction enhances translational potential. The multi-scale approach spanning bulk transcriptomics, AI-driven biomarker selection, single-cell characterization, and functional validation represents an effective paradigm for developing clinically relevant cancer biomarkers and therapeutic targets.

## Introduction

Thyroid cancer represents the most common endocrine malignancy, with its global incidence rising rapidly over the past several decades, largely attributed to improved diagnostic techniques and increased detection of small papillary carcinomas (Chen et al., 2023; Moleti et al., 2023). Despite generally favorable prognosis for well-differentiated thyroid cancers, challenges remain in accurate preoperative diagnosis, risk stratification, and treatment of aggressive or recurrent disease (Nabhan et al., 2021; Borzooei et al., 2024). Current diagnostic approaches rely heavily on fine-needle aspiration biopsy and histopathological examination, which can be inconclusive in approximately 15%–30% of cases, leading to diagnostic uncertainty and potential overtreatment or delayed intervention (Schlumberger et al., 2021). The molecular heterogeneity of thyroid cancer subtypes further complicates clinical management, highlighting the urgent need for robust molecular biomarkers that can enhance diagnostic accuracy and guide personalized therapeutic strategies (Liu et al., 2023; Affinito et al., 2022).

The advent of high-throughput omics technologies has revolutionized biomarker discovery in cancer research, enabling comprehensive profiling of genomic, transcriptomic, and epigenomic alterations associated with tumorigenesis (Zielinski et al., 2021; Wang et al., 2024). Traditional approaches utilizing microarray and RNA-sequencing technologies have successfully identified numerous cancer biomarkers, yet these methods often generate vast datasets that require sophisticated analytical tools for meaningful interpretation (Sun et al., 2021). Traditional bulk sequencing methods measure average gene expression across all cells in a sample, potentially masking critical cell-type-specific patterns and tumor microenvironment dynamics (Li et al., 2022). Single-cell RNA-sequencing technologies have emerged to address this limitation, enabling characterization of cellular heterogeneity, identification of rare cell populations, and mapping of spatial biomarker distributions within the tumor ecosystem (Tan et al., 2023). The integration of bulk and single-cell approaches represents the current frontier in biomarker discovery, providing multi-scale molecular profiling essential for clinical diagnostics and therapeutic development (Moradi Kashkooli et al., 2024).

Meanwhile, artificial intelligence and machine learning approaches have emerged as powerful complementary tools, offering enhanced pattern recognition capabilities and the ability to integrate multi-dimensional data (Dlamini et al., 2020). However, AI-driven discoveries require validation through traditional experimental approaches to ensure biological relevance and mechanistic understanding (Mann et al., 2021). Recent advances in computational biology and machine learning have enabled more sophisticated approaches to biomarker discovery, including meta-analysis frameworks that can integrate multiple independent datasets to enhance statistical power and generalizability (Maleki et al., 2023). When coupled with feature selection algorithms and machine learning models, these approaches can identify optimal biomarker combinations that demonstrate superior performance compared to individual markers (Ligero et al., 2025). However, computational predictions must be rigorously validated through comprehensive experimental approaches to ensure clinical relevance and translational potential (Patel et al., 2022).

MicroRNAs have garnered significant attention as promising diagnostic and therapeutic targets due to their fundamental roles in gene regulation and their remarkable stability in biological fluids (Chorley et al., 2021). These small non-coding RNAs, typically 20–24 nucleotides in length, regulate gene expression post-transcriptionally by binding to complementary sequences in target mRNA molecules, leading to translational repression or mRNA degradation (Wang et al., 2021). Dysregulation of microRNA expression has been implicated in various cancer types, including thyroid cancer, where specific microRNAs function as either oncogenes or tumor suppressors (Ruiz-Pozo et al., 2023). The tissue-specific expression patterns and stability of microRNAs in circulation make them particularly attractive as non-invasive diagnostic biomarkers, while their regulatory functions present opportunities for therapeutic intervention through mimic or antagonist approaches (Oskouie et al., 2022).

Previous studies have identified several microRNAs associated with thyroid cancer, including miR-221, miR-222, miR-146b, and miR-181a, which have shown promise as diagnostic markers and therapeutic targets (Zhu et al., 2022; Lin et al., 2022; Cao et al., 2023; Sun et al., 2022). However, most existing research has focused on well-characterized microRNAs, and comprehensive systematic approaches integrating computational prediction with experimental validation remain limited (Yang et al., 2021). Furthermore, the functional roles of many recently discovered microRNAs in thyroid cancer pathogenesis remain largely unexplored, representing a significant knowledge gap in our understanding of thyroid cancer molecular mechanisms. The integration of traditional omics approaches with AI-driven analytical methods and single-cell resolution validation offers an unprecedented opportunity to systematically identify novel microRNA biomarkers while ensuring robust experimental validation and cell-type specificity essential for clinical translation (Aswathy et al., 2024).

This study addresses the critical need for novel thyroid cancer biomarkers by implementing an integrated approach that combines traditional transcriptomic analysis with AI-driven biomarker discovery methods and single-cell spatial mapping. We hypothesized that systematic meta-analysis of multiple independent datasets, followed by machine learning-based feature selection and single-cell validation, would identify robust biomarker combinations with enhanced diagnostic performance and cell-type specificity. Furthermore, we proposed that comprehensive experimental validation of identified microRNA biomarkers would reveal novel therapeutic targets and provide insights into thyroid cancer molecular mechanisms. Our research aims to bridge the gap between computational biomarker discovery, single-cell characterization, and experimental validation, ultimately advancing both our understanding of thyroid cancer biology and the development of clinically relevant diagnostic and therapeutic tools.

## Methods

### Dataset collection and preprocessing

Three publicly available gene expression datasets (GSE65144, GSE33630, and GSE50901) were downloaded from the Gene Expression Omnibus (GEO) database (https://www.ncbi.nlm.nih.gov/geo/) using the GEOquery package (version 2.60.0) in R software (version 4.3.0; R Foundation for Statistical Computing, Vienna, Austria). GSE65144 and GSE33630 served as training datasets, while GSE50901 was used as an independent validation cohort. Raw expression data were normalized using the robust multi-array average (RMA) algorithm implemented in the affy package (version 1.70.0). Batch effects between datasets were corrected using the ComBat function from the sva package (version 3.40.0). Quality control was performed by principal component analysis and hierarchical clustering to identify potential outliers. Sample annotations were carefully reviewed to ensure accurate classification of tumor and normal samples.

### Meta-analysis and biomarker identification

Meta-analysis was conducted using the MetaIntegrator package (version 2.1.3) in R to identify differentially expressed genes across the training datasets. Effect sizes were calculated using standardized mean differences with 95% confidence intervals. Genes with false discovery rate (FDR) less than 0.05 and absolute effect size greater than 1.5 were considered significantly differentially expressed. Forward feature selection was performed using the stepAIC function from the MASS package (version 7.3–58.1) to identify the optimal biomarker combination. The final four-gene panel consisting of BID, MIR6756, ITM2A, and TGM2 was selected based on the lowest Akaike Information Criterion (AIC) value and cross-validation performance.

### Machine learning model development and validation

A logistic regression model was constructed using the glm function in R with the four-gene biomarker panel as predictors. Model performance was evaluated using receiver operating characteristic (ROC) curve analysis implemented in the pROC package (version 1.18.0). Area under the curve (AUC) values with 95% confidence intervals were calculated for both training datasets and the independent validation dataset. Ten-fold cross-validation was performed to assess model stability and prevent overfitting. Sensitivity, specificity, positive predictive value, and negative predictive value were calculated at the optimal threshold determined by Youden’s index.

### Single-cell RNA-seq data acquisition and processing

Single-cell RNA-sequencing data were obtained from the Gene Expression Omnibus database (accession number GSE184362), encompassing 23 samples from 11 thyroid papillary carcinoma patients. The dataset included seven primary tumor samples, six para-tumor normal tissues, eight lymph node metastasis samples, and two subcutaneous metastasis samples. Raw data in 10X Genomics format (matrix.mtx, barcodes.tsv, and features.tsv files) were downloaded and processed using Seurat package (version 5.0.1) in R (version 4.3.0). Quality control was performed by filtering cells with fewer than 200 or more than 6,000 detected genes, fewer than 500 or more than 50,000 UMI counts, and mitochondrial gene percentages above 20%. After quality control, 196,145 cells were retained and downsampled to 50,000 cells for downstream analysis to optimize computational efficiency while maintaining statistical power. The down sampled dataset-maintained representation from all 23 samples with proportional sampling and previous single-cell studies have demonstrated that 2,000–5,000 cells per condition are typically sufficient for robust differential expression analysis, and our 50,000 cells across 23 samples (average ∼2,174 cells per sample) exceeds this threshold. Data normalization was performed using the LogNormalize method with a scale factor of 10,000. The top 2,000 variable features were identified using the FindVariableFeatures function with the vst selection method. Gene expression data were scaled using the ScaleData function, regressing out the effects of UMI counts and mitochondrial percentage. Principal component analysis was performed using 30 principal components, and the RunPCA function was used with the previously identified variable features. Uniform Manifold Approximation and Projection (UMAP) dimensionality reduction was performed using the RunUMAP function with dimensions 1 through 20. Cells were clustered using the Louvain algorithm implemented in the FindNeighbors and FindClusters functions with resolution parameter set to 0.5, resulting in 27 clusters.

### Cell type annotation and immune microenvironment analysis

Cell type annotation was performed based on expression of canonical marker genes. For each cluster, average expression levels of cell-type-specific markers were calculated, and cell types were assigned based on highest marker expression scores. The marker genes used were as follows: epithelial cells were identified by expression of EPCAM, KRT19, KRT18, TG, and TPO; T cells by CD3D, CD3E, and CD3G; CD8-positive T cells by CD8A and CD8B; CD4-positive T cells by CD4 and IL7R; regulatory T cells by FOXP3 and IL2RA; B cells by CD79A, MS4A1, and CD19; plasma cells by JCHAIN, MZB1, and SDC1; myeloid cells by LYZ, CD14, and CD68; macrophages by CD68, CD163, and MSR1; dendritic cells by CLEC9A, CLEC10A, and CD1C; natural killer cells by GNLY, NKG7, and KLRD1; fibroblasts by COL1A1, COL1A2, and DCN; and endothelial cells by PECAM1, VWF, and CDH5. Immune microenvironment composition was quantified by calculating the proportion of immune cell subtypes across different sample groups. CD8-positive to CD4-positive T cell ratios were calculated as indicators of cytotoxic immune activity. Statistical comparisons of cell type proportions between metastatic and non-metastatic sample groups were performed using chi-square tests with Bonferroni correction for multiple comparisons.

### Biomarker expression analysis in single-cell data

Expression patterns of the identified biomarkers (BID, ITM2A, and TGM2) were examined across all cell types in the single-cell dataset. For microRNA analysis, expression of hsa-miR-6756-5p was evaluated where available in the dataset. Mean expression levels were calculated for each cell type and sample group using the GetAssayData function to access the normalized expression matrix. Cell-type-specific expression patterns were visualized using UMAP plots with expression values color-coded using the viridis color scale, and heatmaps showing average expression across cell types and metastatic status were generated using the ggplot2 package (version 3.4.0). Statistical comparisons of biomarker expression between metastatic and non-metastatic samples were performed using Wilcoxon rank-sum tests with Bonferroni correction for multiple testing.

### Survival analysis and correlation studies

Survival analysis was performed using clinical data from The Cancer Genome Atlas (TCGA) thyroid carcinoma cohort downloaded through the TCGAbiolinks package (version 2.26.0). Patients were stratified into high and low expression groups based on the median expression value of each gene. Kaplan-Meier survival curves were constructed using the survminer package (version 0.4.9) and compared using the log-rank test. Hazard ratios with 95% confidence intervals were calculated using Cox proportional hazards regression models implemented in the survival package (version 3.5–0). Correlation analysis between microRNA and mRNA expression levels was performed using Spearman’s rank correlation coefficient, with statistical significance defined as p less than 0.05.

### Cell culture and maintenance

Human thyroid cancer cell lines TPC-1 and BHT101 were obtained from the American Type Culture Collection (ATCC, Manassas, VA, USA; catalog numbers CRL-3365 and HTB-104, respectively). TPC-1 cells were cultured in Dulbecco’s Modified Eagle Medium (DMEM; catalog number 11965092, Gibco, Thermo Fisher Scientific, Waltham, MA, USA) supplemented with 10% fetal bovine serum (FBS; catalog number 10270106, Gibco), 100 units per milliliter penicillin, and 100 μg per milliliter streptomycin (catalog number 15140122, Gibco). BHT101 cells were maintained in RPMI-1640 medium (catalog number 11875093, Gibco) with 10% FBS and antibiotics. All cells were incubated at 37 °C in a humidified atmosphere containing 5% carbon dioxide using a Thermo Scientific Heracell VIOS 160i CO2 incubator (Thermo Fisher Scientific). Cell lines were authenticated by short tandem repeat profiling performed by ATCC and tested for *mycoplasma* contamination using the MycoAlert *Mycoplasma* Detection Kit (catalog number LT07-318, Lonza, Basel, Switzerland).

### Stable vector construction for hsa-miR-6756-5p overexpression

For stable overexpression of has-miR-6756-5p, the primary transcript (pri-miR-6756) was cloned into the pCDH-CMV-MCS-EF1α-Puro lentiviral vector (catalog number CD510B-1, System Biosciences, Palo Alto, CA, USA). The pri-miR-6756 sequence including 200 base pairs upstream and downstream genomic flanking regions was synthesized as a gBlock Gene Fragment (Integrated DNA Technologies, Coralville, IA, USA) with EcoRI and BamHI restriction sites. The vector and insert were digested using EcoRI-HF and BamHI-HF restriction enzymes (catalog numbers R3101S and R3136S, respectively, New England Biolabs, Ipswich, MA, USA) at 37 °C for 2 h. Ligation was performed using T4 DNA Ligase (catalog number M0202S, New England Biolabs) at 16 °C overnight with a vector to insert molar ratio of one–3. The ligation product was transformed into DH5α competent cells (catalog number 18265017, Invitrogen, Carlsbad, CA, USA), and positive clones were selected on Luria-Bertani agar plates containing 100 μg per milliliter ampicillin. Clones were verified by Sanger sequencing performed by Genewiz (South Plainfield, NJ, USA) using CMV forward primer (5′-CGC​AAA​TGG​GCG​GTA​GGC​GTG-3′) and EF1α reverse primer (5′-GTG​TGG​AAA​GTC​CCC​AGG​CT-3′).

### CRISPRi-mediated has-miR-6756-5p knockdown system

For stable knockdown of miR-6756-5p, a CRISPR interference system utilizing catalytically inactive Cas9 fused to the KRAB repressor domain was employed. The lentiviral vector pLV-EF1α-dCas9-KRAB-T2A-Puro (Addgene plasmid number 71236, Addgene, Watertown, MA, USA) was used to express the dCas9-KRAB fusion protein. Guide RNAs targeting the MIR6756 promoter region were designed using the CHOPCHOP web tool (http://chopchop.cbu.uib.no/) and cloned into the pLKO.1-U6-sgRNA-EF1α-Puro vector (Addgene plasmid number 60958). Two guide RNA sequences were designed: sgRNA-1 with sequence 5′-GCA​GCT​ACT​GCA​GGC​ATC​TG-3′ and sgRNA-2 with sequence 5′-TGC​AGG​CAT​CTG​TGG​CTA​AG-3'. Oligonucleotides were synthesized by Integrated DNA Technologies and annealed before ligation into BsmBI-digested vector using T4 DNA Ligase. A non-targeting control sgRNA with the sequence 5′-GTA​GCG​AAC​GTG​TCC​GGC​GT-3′ was used as a negative control and is referred to as CRISPRi-NC throughout the manuscript.

### Lentiviral production and transduction

Lentiviral particles were produced by co-transfecting HEK293T cells (catalog number CRL-3216, ATCC) with the transfer vector, psPAX2 packaging plasmid (Addgene plasmid number 12260), and pMD2.G envelope plasmid (Addgene plasmid number 12259) using Lipofectamine 3000 Transfection Reagent (catalog number L3000015, Invitrogen) according to the manufacturer’s protocol. The transfection ratio was four to three to one for transfer vector to packaging plasmid to envelope plasmid. Culture medium was replaced with fresh DMEM containing 2% FBS after 6 h. Viral supernatants were collected at 48- and 72-h post-transfection, filtered through 0.45-μm cellulose acetate syringe filters (catalog number SLHA033SS, Millipore, Burlington, MA, USA), and concentrated using Lenti-X Concentrator (catalog number 631231, Takara Bio, Kusatsu, Japan). Target cells were infected with concentrated lentivirus in the presence of 8 μg per milliliter polybrene (catalog number TR-1003-G, Sigma-Aldrich, St. Louis, MO, USA) at a multiplicity of infection of 5–10. Stable cell lines were selected using 2 μg per milliliter puromycin (catalog number P8833, Sigma-Aldrich) starting 48 h post-infection and maintained in selection medium for 2 weeks.

### Transient transfection with miRNA mimics and inhibitors

For transient modulation of miR-6756-5p expression, cells were transfected with miR-6756-5p mimic, inhibitor, or corresponding negative controls (GenePharma, Shanghai, China) using Lipofectamine RNAiMAX Transfection Reagent (catalog number 13778075, Invitrogen). The miR-6756-5p mimic had the sequence 5′-AGG​GUG​GGG​CUG​GAG​GUG​GGG​CU-3′, and the inhibitor was a chemically modified 2′-O-methyl antisense oligonucleotide complementary to the mature miR-6756-5p sequence with phosphorothioate backbone modifications. Cells were seeded in 6-well plates (catalog number 353046, Corning, Corning, NY, USA) at 60 to 70 percent confluency 24 h before transfection. Transfections were performed with 50 nM mimic or 100 nM inhibitor according to the manufacturer’s instructions. Negative control mimic (NC) and inhibitor (anti-NC) were used as controls. Transfection efficiency was monitored using FAM-labeled control oligonucleotides, and experiments were performed 24–48 h post-transfection.

### RNA extraction and quantitative Real-Time PCR

Total RNA was extracted using TRIzol Reagent (catalog number 15596026, Invitrogen) according to the manufacturer’s protocol. RNA concentration and purity were determined using a NanoDrop 2000 spectrophotometer (Thermo Fisher Scientific), with A260/A280 ratios between 1.8 and 2.0 considered acceptable. For microRNA quantification, reverse transcription was performed using the miScript II RT Kit (catalog number 218161, Qiagen, Hilden, Germany) with 1 μg total RNA in a 20-μL reaction volume. Quantitative PCR was conducted using the miScript SYBR Green PCR Kit (catalog number 218073, Qiagen) on a QuantStudio 3 Real-Time PCR System (catalog number A28137, Applied Biosystems, Foster City, CA, USA). The miR-6756-5p-specific primer was obtained from Qiagen (catalog number MS00031240), and U6 small nuclear RNA was used as an endogenous control (catalog number MS00033740, Qiagen). PCR cycling conditions were: 95 °C for 15 min, followed by 40 cycles of 94 °C for 15 s, 55 °C for 30 s, and 70 °C for 30 s. For mRNA quantification, complementary DNA synthesis was performed using the High-Capacity cDNA Reverse Transcription Kit (catalog number 4368814, Applied Biosystems), and quantitative PCR was conducted using PowerUp SYBR Green Master Mix (catalog number A25742, Applied Biosystems) with gene-specific primers synthesized by Integrated DNA Technologies. GAPDH was used as an endogenous control with primers: forward 5′-GTC​TCC​TCT​GAC​TTC​AAC​AGC​G-3′ and reverse 5′-ACC​ACC​CTG​TTG​CTG​TAG​CCA​A-3'. Relative expression levels were calculated using the two to the negative delta-delta Ct method.

#### Cell proliferation assays

Cell proliferation was assessed using the Cell Counting Kit-8 (CCK-8; catalog number CK04, Dojindo Laboratories, Kumamoto, Japan). Cells were seeded in 96-well plates (catalog number 353072, Corning) at a density of 2,000 cells per well in triplicate. At designated time points (24, 48, 72, and 96 h), 10 μL of CCK-8 solution was added to each well, and plates were incubated for 2 h at 37 °C. Absorbance was measured at 450 nm using a Synergy H1 Hybrid Multi-Mode Microplate Reader (BioTek Instruments, Winooski, VT, USA). Growth curves were plotted using GraphPad Prism software version 9.0 (GraphPad Software, San Diego, CA, USA), and area under the curve was calculated to quantify proliferation differences between experimental groups. For EdU incorporation assays, cells were incubated with 10 μM five-ethynyl-2′-deoxyuridine (EdU; catalog number C10337, Invitrogen) for 2 h before fixation with 4% paraformaldehyde and detection using the Click-iT EdU Alexa Fluor 488 Imaging Kit (catalog number C10337, Invitrogen) according to the manufacturer’s instructions. Images were acquired using an EVOS FL Auto 2 Imaging System (Thermo Fisher Scientific), and EdU-positive cells were quantified using ImageJ software version 1.53 (National Institutes of Health, Bethesda, MD, USA).

#### Colony formation assays

For clonogenic assays, cells were seeded in 6-well plates at a density of 500 to 1,000 cells per well depending on the cell line. Cells were cultured for 10–14 days with medium changes every 3–4 days until visible colonies formed. Colonies were fixed with 4% paraformaldehyde (catalog number 158127, Sigma-Aldrich) for 15 min at room temperature and stained with 0.1% crystal violet solution (catalog number C0775, Sigma-Aldrich) for 30 min. After washing with distilled water, colonies containing more than 50 cells were counted under an inverted microscope (CKX53, Olympus, Tokyo, Japan). Colony formation efficiency was calculated as the percentage of seeded cells that formed colonies. Images were captured using a Canon EOS 600D digital camera (Canon, Tokyo, Japan) mounted on the microscope, and colony areas were quantified using ImageJ software.

#### Cell migration and invasion assays

Cell migration was evaluated using Transwell chambers with 8-μm pore size polycarbonate membrane inserts (catalog number 3422, Corning). Cells were serum-starved for 12 h before seeding 50,000 cells in 200 μL serum-free medium in the upper chamber. The lower chamber contained 600 μL complete medium with 10% FBS as a chemoattractant. After 24 h incubation at 37 °C, non-migrated cells on the upper surface were removed with cotton swabs, and migrated cells were fixed with 4% paraformaldehyde for 15 min and stained with 0.1% crystal violet for 20 min. For invasion assays, Transwell inserts were pre-coated with 50 μL Matrigel Matrix (catalog number 356234, Corning) diluted one to three in serum-free medium and incubated at 37 °C for 2 h before cell seeding. Cells were incubated for 48 h to allow invasion through the Matrigel barrier. Migrated and invaded cells were photographed using the Olympus CKX53 inverted microscope with a mounted Canon digital camera, and cells were counted in five random fields per insert at 200-times magnification. Cell numbers were quantified using ImageJ software, and statistical analysis was performed using GraphPad Prism.

### Wound healing scratch assays

Cells were seeded in 6-well plates and grown to 90 to 95 percent confluency. A sterile 200-μL pipette tip was used to create straight scratches across the cell monolayer. Cells were washed twice with phosphate-buffered saline (PBS; catalog number 14190144, Gibco) to remove debris and cultured in serum-free medium to minimize proliferation effects. Scratch wounds were photographed immediately after creation (0 h) and at 24 and 48 h using the Olympus CKX53 inverted microscope equipped with the Canon digital camera. Wound closure was quantified by measuring the scratch area using ImageJ software. The percentage of wound closure was calculated as: [(initial wound area minus final wound area) divided by initial wound area] times 100%. Experiments were performed in triplicate with at least three different scratch areas measured per well. Statistical analysis was performed using two-way repeated measures ANOVA in GraphPad Prism.

### *In Vivo* xenograft experiments

Animal experiments were conducted in accordance with institutional guidelines and approved by the Institutional Animal Care and Use Committee (approval number 2021–1407). Six-week-old male BALB/c nude mice weighing 18–22 g were purchased from Charles River Laboratories (Wilmington, MA, USA; strain code 028) and maintained in a pathogen-free environment with a 12-h light-dark cycle, controlled temperature (22–24 °C), and humidity (40–60 percent). Mice were randomly divided into experimental groups with five mice per group. TPC-1 cells (2 times 10 to the power of 6) stably expressing miR-6756-5p, CRISPRi-mediated knockdown constructs, or corresponding controls were suspended in 100 μL PBS and injected subcutaneously into the right flank of each mouse using a 1-mL syringe with a 26-gauge needle. Tumor growth was monitored every 3 days using digital calipers (catalog number 14–648-17, Fisher Scientific, Hampton, NH, USA), and tumor volume was calculated using the formula: volume equals (length times width squared) divided by 2, where length is the longest dimension and width is the perpendicular measurement. Mice were euthanized when tumors reached 1,500 cubic millimeters or at the end of the 27-day observation period by carbon dioxide inhalation followed by cervical dislocation. Tumors were excised, weighed using an analytical balance (model AX224, Ohaus Corporation, Parsippany, NJ, USA), photographed using a Canon digital camera, and stored at minus 80 °C for further analysis.

### Statistical analysis

All experiments were performed with at least three independent biological replicates unless otherwise specified. Data are presented as mean plus or minus standard error of the mean (SEM). Statistical analyses were conducted using GraphPad Prism software version 9.0 and R statistical software version 4.3.0. Comparisons between two groups were performed using unpaired two-tailed Student's t-tests for normally distributed data or Mann-Whitney U tests for non-parametric data. Normality of data distribution was assessed using the Shapiro-Wilk test. Multiple group comparisons were analyzed using one-way analysis of variance (ANOVA) followed by Tukey’s *post hoc* test for normally distributed data or Kruskal–Wallis test followed by Dunn’s *post hoc* test for non-parametric data. For time-course experiments, two-way repeated measures ANOVA was used with Sidak’s *post hoc* test for multiple comparisons. Survival curves were compared using the log-rank (Mantel-Cox) test. Statistical significance was defined as p less than 0.05, with specific p-values indicated in figure legends using the following notation: asterisk indicates p less than 0.05, double asterisk indicates p less than 0.01, and triple asterisk indicates p less than 0.001. Effect sizes and 95% confidence intervals were reported where appropriate.

## Results

### Identification of Four-Gene Biomarker Panel Through Meta-Analysis and Machine Learning approaches

**FIGURE 1:**
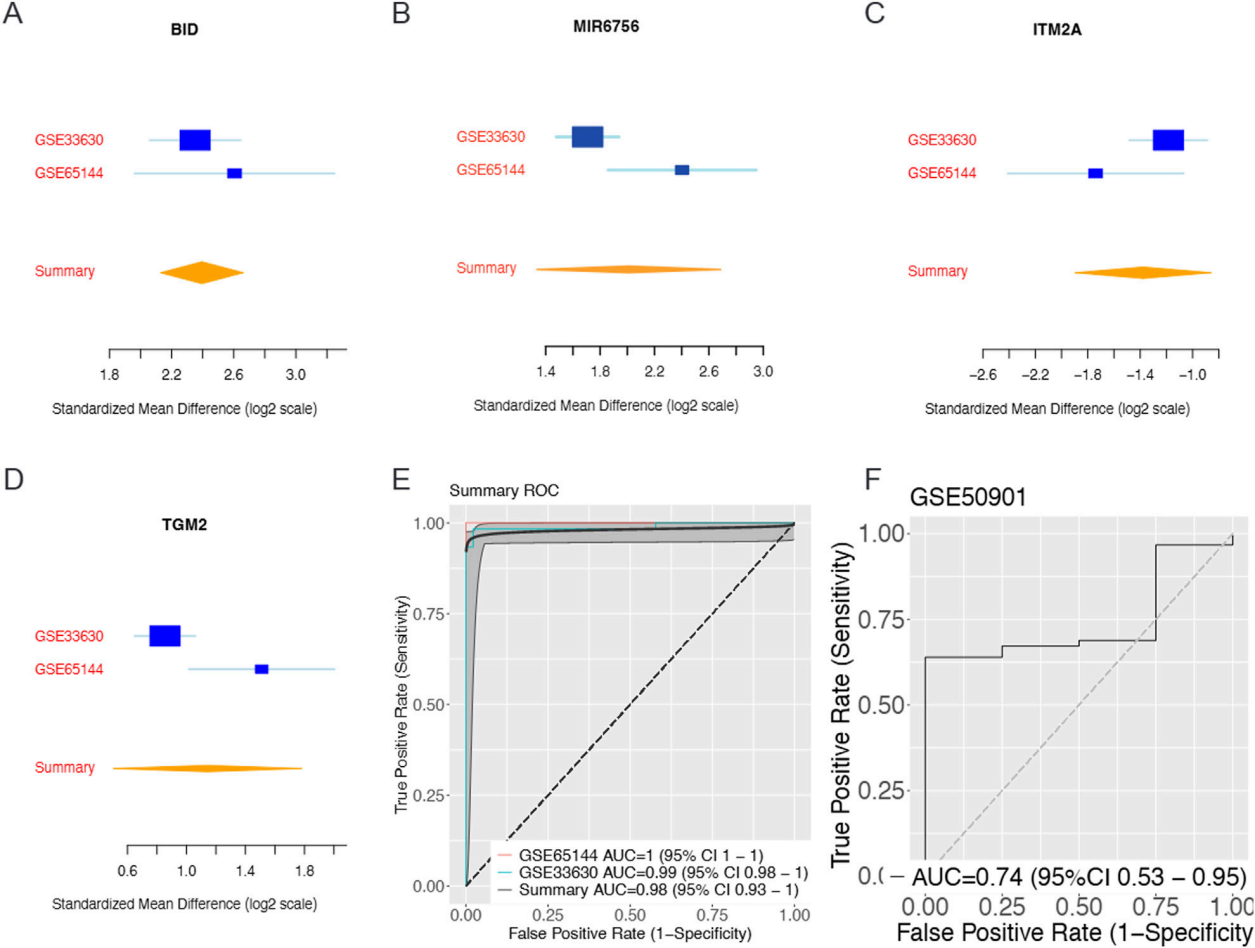
Identification and Validation of Four-Gene Biomarker Panel Through Meta-Analysis and Machine Learning. Meta-analysis of gene expression datasets reveals a robust four-gene biomarker panel for thyroid cancer diagnosis. Forest plots showing standardized mean differences (log2 scale) with 95% confidence intervals for **(A)** BID, **(B)** ITM2A, **(C)** TGM2, and **(D)** MIR6756 across training datasets GSE33630 and GSE65144, with summary effect sizes calculated using random-effects meta-analysis. BID and MIR6756 demonstrate consistent upregulation in thyroid cancer samples, while ITM2A shows significant downregulation and TGM2 exhibits moderate upregulation. **(E)** Receiver operating characteristic (ROC) curves for the four-gene panel showing area under the curve (AUC) values of 1.0 (95% CI: 1.0–1.0) for GSE65144 and 0.99 (95% CI: 0.98–1.0) for GSE33630, with summary AUC of 0.98 (95% CI: 0.93–1.0). **(F)** Independent validation in dataset GSE50901 achieved AUC of 0.74 (95% CI: 0.53–0.95), demonstrating good generalizability of the biomarker panel across different populations and technical platforms.

To identify robust biomarkers for thyroid cancer diagnosis, we performed a comprehensive meta-analysis using three independent gene expression datasets: GSE65144, GSE33630 (training sets), and GSE50901 (validation set). Through systematic application of meta-integrator effect size analysis followed by forward feature selection algorithms, we identified a four-gene biomarker panel consisting of BID, MIR6756, ITM2A, and TGM2. The standardized mean differences revealed distinct expression patterns for each biomarker across the datasets (Figures 1A–D). BID and MIR6756 demonstrated consistent upregulation in thyroid cancer samples, while ITM2A showed significant downregulation. TGM2 exhibited moderate but consistent upregulation across all datasets. The machine learning model constructed using this four-gene panel achieved exceptional discriminatory performance with area under the curve (AUC) values of 1.0 (95% CI: 1.0–1.0) in GSE65144 and 0.99 (95% CI: 0.98–1.0) in GSE33630 (Figure 1E). Importantly, the model maintained good generalizability in the independent validation dataset GSE50901, achieving an AUC of 0.74 (95% CI: 0.53–0.95) (Figure 1F)

## MicroRNA-6756 mature forms show differential expression and Clinical Associations

**FIGURE 2:**
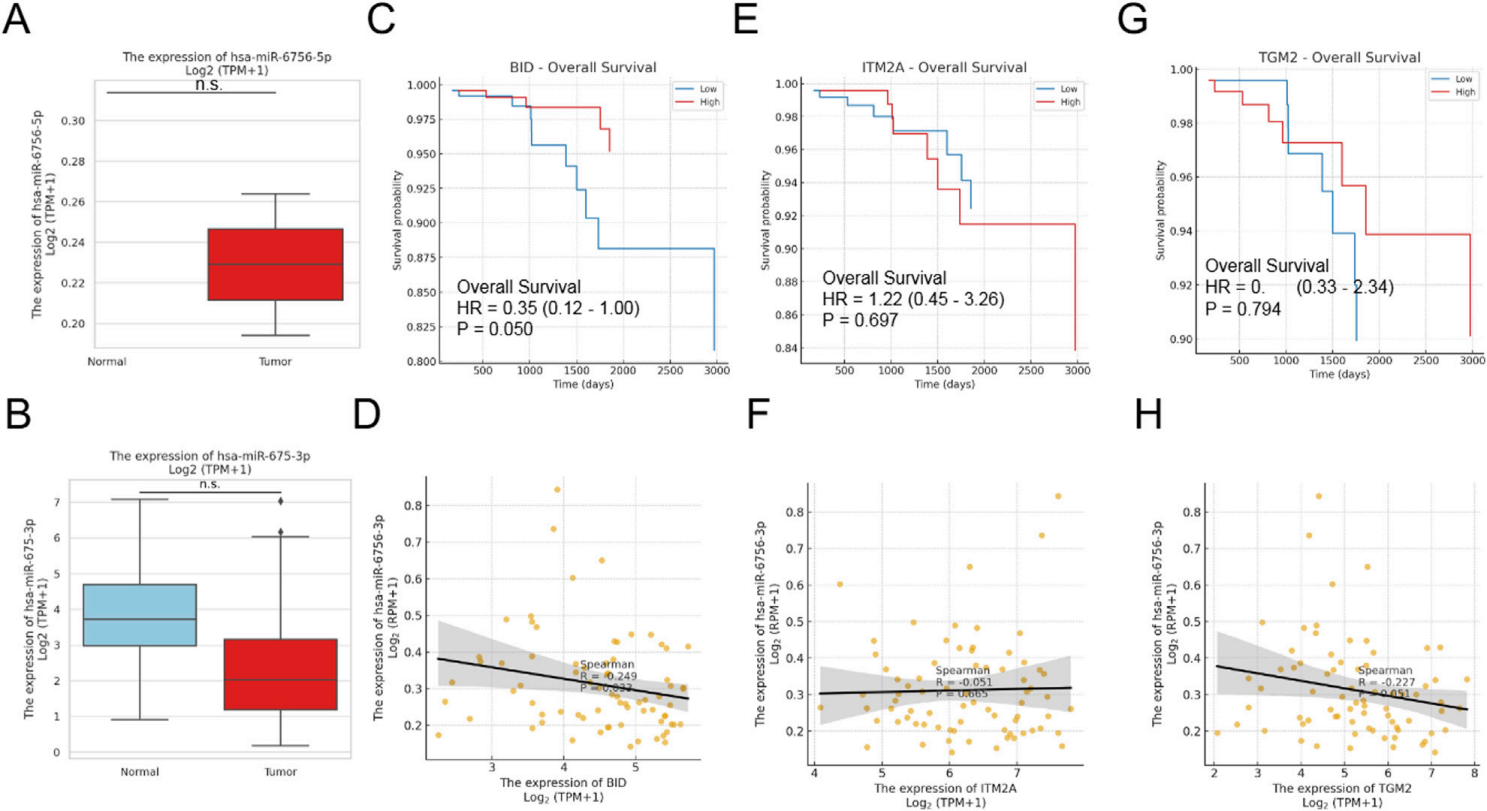
MicroRNA-6756 Expression Patterns and Clinical Associations in Thyroid Cancer. Analysis of microRNA-6756 mature forms reveals distinct expression patterns and clinical correlations. **(A)** Expression levels of hsa-miR-6756-5p (upper panel) and hsa-miR-6756-3p (lower panel) in thyroid cancer versus normal tissues from TCGA dataset, showing tumor-specific expression of the 5p form while the 3p form exhibits no significant differential expression. **(B)** Additional validation of hsa-miR-6756-3p expression in normal versus tumor samples confirms lack of significant difference between groups. Kaplan-Meier survival curves for **(C)** BID, **(D)** ITM2A, and **(E)** TGM2 expression in thyroid cancer patients, with only BID showing significant association with overall survival (hazard ratio equals 0.35, 95% CI: 0.12–1.00, p equals 0.050). Correlation analysis between hsa-miR-6756-3p expression and **(F)** BID (Spearman R equals negative 0.249, p equals 0.033), **(G)** ITM2A (Spearman R equals negative 0.051, p equals 0.665), and **(H)** TGM2 (Spearman R equals negative 0.227, p equals 0.052) reveals weak negative correlations that do not reach statistical significance for most comparisons.

Given the identification of MIR6756 as a key biomarker, we investigated the expression patterns of its two mature forms: hsa-miR-6756-5p and hsa-miR-6756-3p. Remarkably, hsa-miR-6756-5p exhibited tumor-specific expression, being virtually absent in normal thyroid tissues but significantly elevated in thyroid cancer samples (Figure 2A, upper panel). This unique expression pattern suggests its potential as a highly specific diagnostic biomarker. In contrast, hsa-miR-6756-3p showed no significant differential expression between tumor and normal tissues (Figure 2A, lower panel). Survival analysis revealed that among the identified biomarkers, only BID expression was significantly associated with overall survival outcomes, with high BID expression correlating with improved survival (hazard ratio equals 0.35, 95% CI: 0.12–1.00, p equals 0.050) (Figures 2C–E). Correlation analysis between the three protein-coding genes and hsa-miR-6756-3p revealed weak negative correlations that did not reach statistical significance, suggesting complex regulatory relationships that may require further investigation (Figures 2F–H).

### Single-cell RNA-seq analysis reveals cellular heterogeneity and immune microenvironment in thyroid cancer

**FIGURE 3:**
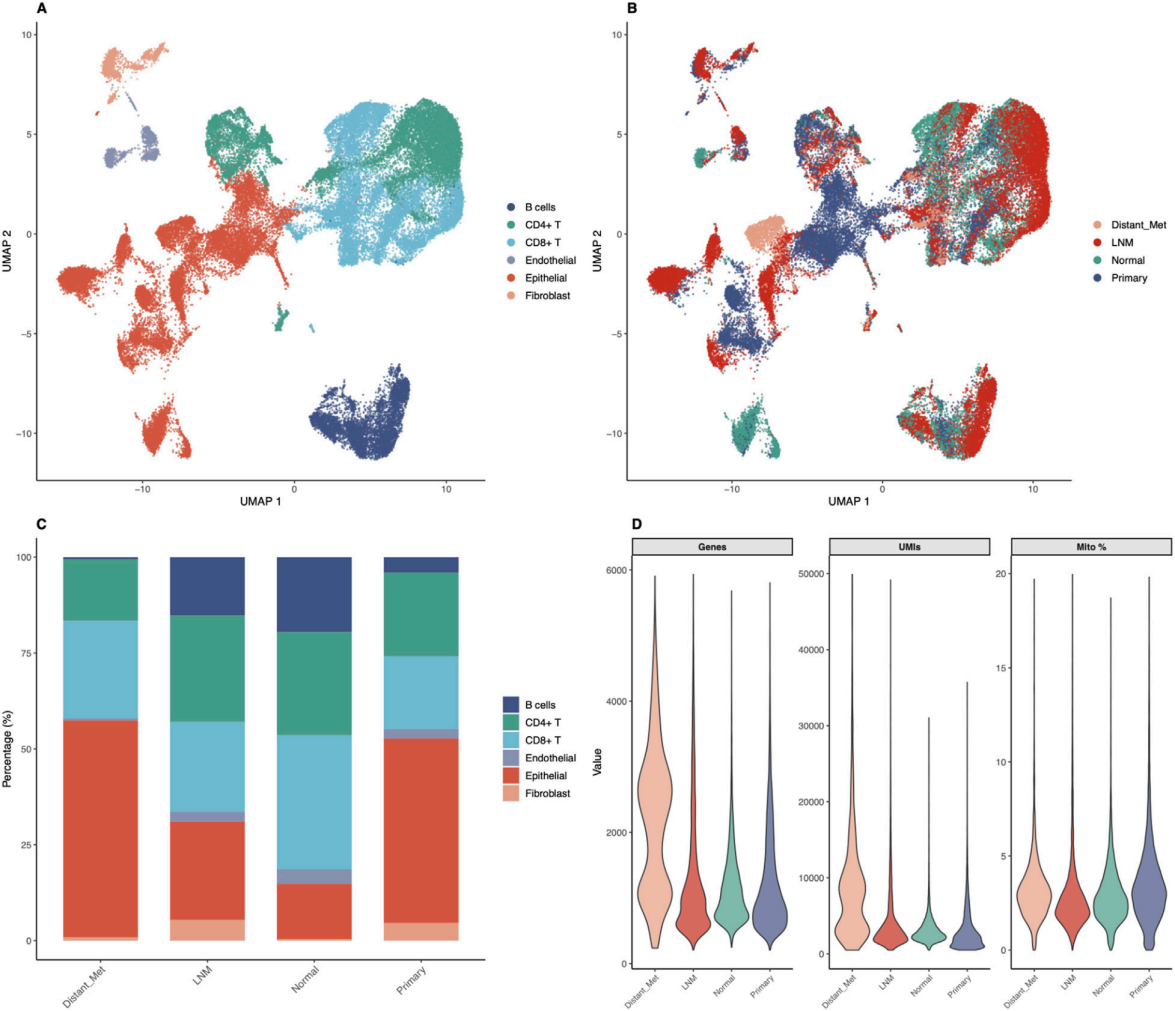
Single-Cell RNA-seq Analysis Reveals Cellular Heterogeneity in Thyroid Cancer. Comprehensive single-cell analysis of 50,000 cells from 23 thyroid cancer samples demonstrates cellular diversity and tumor microenvironment composition. **(A)** UMAP visualization colored by cell type annotation, showing six major populations: Epithelial cells (31.8%, 15,878 cells), CD8-positive T cells (25.1%, 12,552 cells), CD4-positive T cells (24.9%, 12,471 cells), B cells (11.9%, 5,933 cells), Fibroblasts (3.6%, 1,795 cells), and Endothelial cells (2.7%, 1,371 cells). Immune cells comprise 61.9% of total analyzed cells. **(B)** UMAP colored by metastatic status (Lymph Node Metastasis, Normal, Primary Tumor, Distant Metastasis) demonstrating differential spatial distribution of sample origins. **(C)** Stacked bar chart showing cell type composition across metastatic groups, revealing proportional differences in cellular populations between different disease stages and sample types. **(D)** Violin plots showing quality control metrics (genes detected per cell, UMI counts per cell, mitochondrial percentage) across sample groups, demonstrating high-quality data with median values of 1,019 genes per cell, 2,711 UMI counts per cell, and 3.36% mitochondrial reads.

To validate our biomarkers at single-cell resolution and characterize the tumor microenvironment, we analyzed publicly available single-cell RNA-sequencing data (GSE184362) comprising 196,145 quality-controlled cells from 23 thyroid cancer samples. Down-sampling to 50,000 cells enabled comprehensive analysis while maintaining statistical power. Cell type annotation based on canonical marker genes identified six major cell populations (Figure 3A). Epithelial cells comprised 15,878 cells (31.8 percent), CD8-positive T cells comprised 12,552 cells (25.1 percent), CD4-positive T cells comprised 12,471 cells (24.9 percent), B cells comprised 5,933 cells (11.9 percent), fibroblasts comprised 1,795 cells (3.6 percent), and endothelial cells comprised 1,371 cells (2.7 percent). Remarkably, immune cells comprised 61.9 percent of total cells (30,956 cells), highlighting the significant immune infiltration in thyroid cancer microenvironment. UMAP visualization revealed distinct clustering patterns for each cell type, with clear separation between epithelial and immune compartments (Figure 3A). Sample stratification by metastatic status showed differential distribution patterns, with metastatic samples displaying altered cellular compositions compared to primary tumors and normal tissues (Figure 3B). The cell type composition across different sample groups revealed interesting patterns (Figure 3C). Quality control metrics demonstrated high-quality data with median values of 1,019 genes per cell, 2,711 UMI counts per cell, and 3.36 percent mitochondrial reads, indicating minimal cellular stress or damage (Figure 3D).

### Cell-type-specific expression patterns of identified biomarkers revealed by single-cell analysis

**FIGURE 4:**
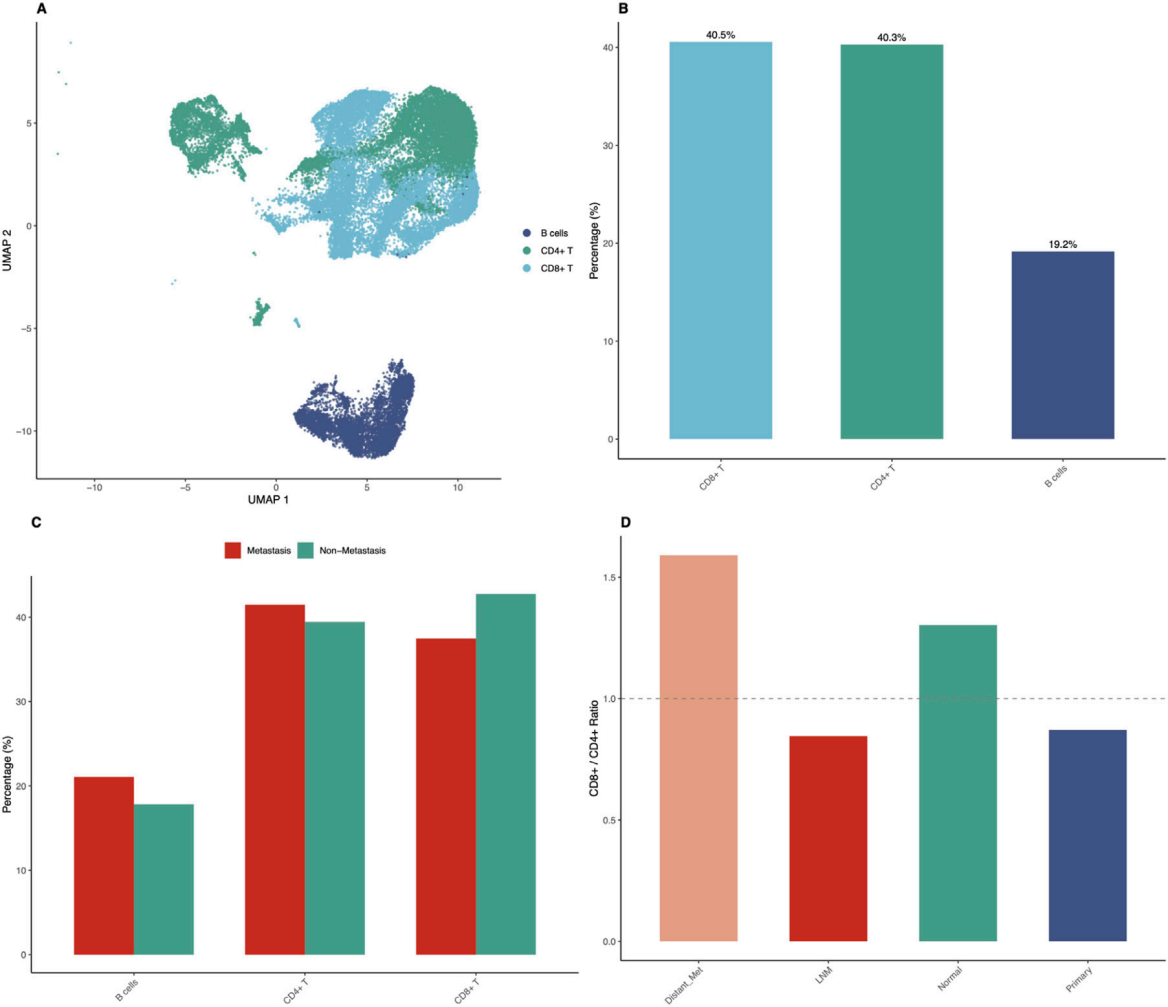
Cell-Type-Specific Expression of Identified Biomarkers at Single-Cell Resolution. Single-cell analysis reveals distinct cell-type-specific expression patterns for the identified biomarkers. UMAP plots showing expression distribution of **(A)** BID, **(B)** ITM2A, and **(C)** TGM2 across all 50,000 cells, with color intensity indicating normalized expression levels using the viridis color scale. BID shows predominant epithelial cell expression with moderate immune cell expression. ITM2A demonstrates highest expression in epithelial cells with lower stromal expression, consistent with its tumor suppressor function. TGM2 exhibits enriched expression in immune cells (particularly macrophages and activated T cells) and fibroblasts, suggesting roles in immune regulation and extracellular matrix remodeling. **(D)** Heatmap showing mean expression of all three biomarkers across cell types stratified by metastatic status (LNM, Normal, Primary, Distant Metastasis), revealing cell-type-specific and condition-dependent expression patterns with differential regulation between metastatic and non-metastatic samples.

Single-cell resolution analysis revealed cell-type-specific expression patterns for our identified biomarkers (Figures 4A–C). BID showed predominantly epithelial cell expression with moderate levels in immune cells, consistent with its role in apoptosis regulation across multiple cell types. The UMAP visualization demonstrated higher BID expression in epithelial cell clusters, with scattered expression in immune and stromal cells. ITM2A demonstrated highest expression in epithelial cells with lower levels in stromal populations, supporting its tumor suppressor function primarily in the epithelial compartment. The spatial distribution of ITM2A expression on the UMAP plot showed clear enrichment in epithelial cell regions. Notably, TGM2 exhibited enriched expression in both immune cells, particularly macrophages and activated T cells, and fibroblasts, suggesting roles in immune regulation and extracellular matrix remodeling beyond epithelial transformation. Expression heatmaps stratified by cell type and metastatic status revealed that all three biomarkers showed differential expression patterns between metastatic and non-metastatic samples across multiple cell types (Figure 4D). This multi-cellular involvement supports their utility as comprehensive biomarkers reflecting tumor ecosystem changes rather than epithelial-specific alterations alone. The heatmap demonstrated that BID expression was elevated in epithelial cells from metastatic samples compared to normal tissues, ITM2A showed reduced expression in tumor-associated epithelial cells, and TGM2 exhibited increased expression in immune and stromal compartments of metastatic samples.

### Immune microenvironment profiling reveals metastasis-associated patterns

**FIGURE 5:**
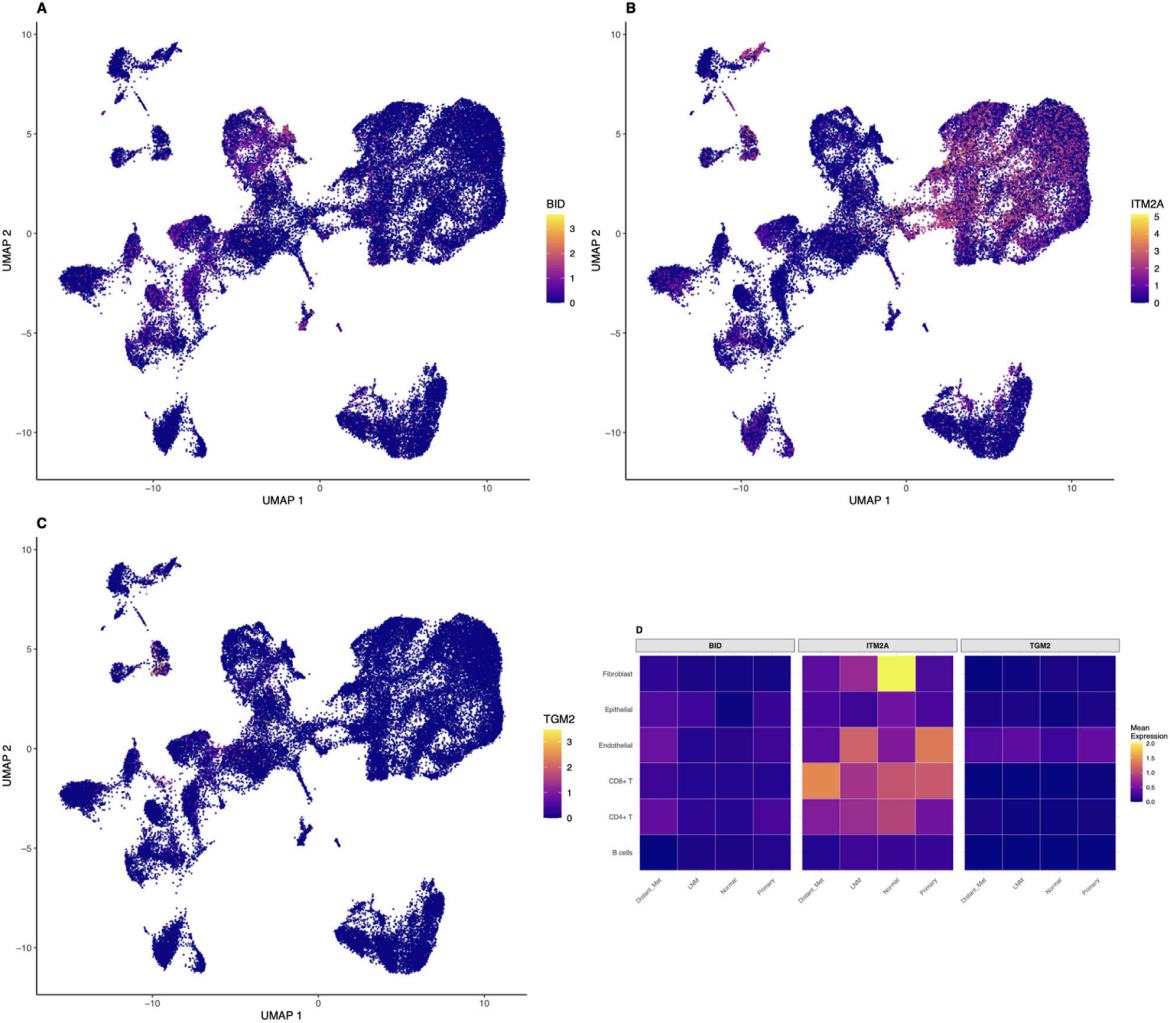
Immune Microenvironment Profiling Reveals Metastasis-Associated Patterns. Detailed characterization of immune cell populations demonstrates significant differences between metastatic and non-metastatic samples. **(A)** UMAP visualization showing only immune cells (CD8-positive T, CD4-positive T, B cells), representing 61.9% of total analyzed cells (30,956 cells), with clear separation of immune cell subtypes. **(B)** Bar chart showing proportions of major immune cell subtypes in the overall dataset: CD8-positive T cells (25.1%), CD4-positive T cells (24.9%), and B cells (11.9%). **(C)** Grouped bar chart comparing immune cell composition between metastatic (LNM plus Distant Metastasis) and non-metastatic (Primary plus Normal) samples, showing differential CD8-positive T cell, CD4-positive T cell, and B cell proportions. Distant metastasis shows 60.6% CD8-positive T, 38.1% CD4-positive T, and 1.3% B cells; LNM shows 35.3% CD8-positive T, 41.8% CD4-positive T, and 22.9% B cells; Normal shows 43.0% CD8-positive T, 33.0% CD4-positive T, and 24.0% B cells; Primary shows 42.4% CD8-positive T, 48.6% CD4-positive T, and 9.0% B cells. **(D)** Bar chart showing CD8-positive to CD4-positive T cell ratios across sample groups, with distant metastases showing elevated ratio (1.59) compared to primary tumors (0.87) and lymph node metastases (0.84).

Detailed immune microenvironment characterization revealed striking differences between metastatic and non-metastatic samples (Figure 5). UMAP visualization of immune cells only, representing 61.9 percent of total analyzed cells, showed clear separation of immune cell subtypes including CD8-positive T cells, CD4-positive T cells, and B cells (Figure 5A). The proportions of immune cell subtypes in the overall dataset showed CD8-positive T cells at 25.1 percent, CD4-positive T cells at 24.9 percent, and B cells at 11.9 percent as major populations (Figure 5B). Immune cell composition analysis across sample groups showed distinct patterns. Distant metastasis samples showed CD8-positive T cells at 60.6 percent, CD4-positive T cells at 38.1 percent, and B cells at only 1.3 percent. Lymph node metastasis samples showed CD8-positive T cells at 35.3 percent, CD4-positive T cells at 41.8 percent, and B cells at 22.9 percent. Normal tissue showed CD8-positive T cells at 43.0 percent, CD4-positive T cells at 33.0 percent, and B cells at 24.0 percent. Primary tumor samples showed CD8-positive T cells at 42.4 percent, CD4-positive T cells at 48.6 percent, and B cells at 9.0 percent (Figure 5C). The CD8-positive to CD4-positive T cell ratio analysis revealed elevated ratios in distant metastatic samples (1.59) compared to primary tumors (0.87) and lymph node metastases (0.84), suggesting enhanced cytotoxic immune responses in advanced disease (Figure 5D). Interestingly, B cell proportions were substantially reduced in distant metastases (1.3 percent) compared to lymph node metastases (22.9 percent) and normal tissues (24.0 percent).

### Functional validation demonstrates oncogenic role of hsa-miR-6756-5p in thyroid cancer cell lines

**FIGURE 6:**
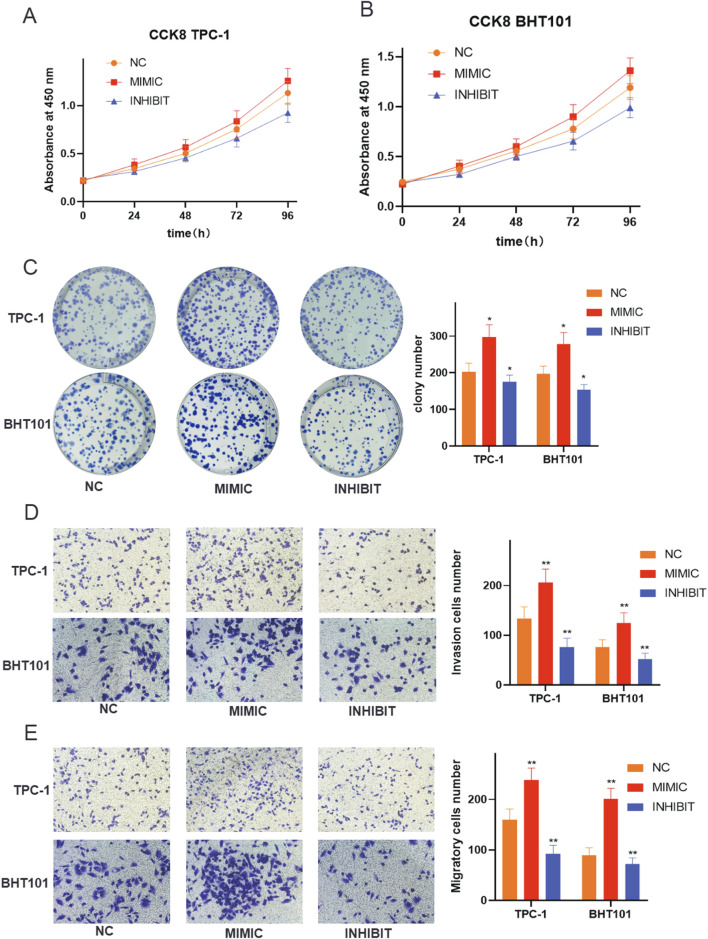
Functional Validation of miR-6756-5p in Thyroid Cancer Cell Lines. Comprehensive functional assays demonstrate the oncogenic properties of miR-6756-5p. Cell proliferation analysis using CCK-8 assays in **(A)** TPC-1 and **(B)** BHT101 cell lines transfected with negative control (NC), miR-6756-5p mimic (MIMIC), or miR-6756-5p inhibitor (INHIBIT) measured at 24, 48, 72, and 96 h, showing significant promotion of cell growth by mimic treatment and inhibition by antagonist treatment in both cell lines. Growth curves show time-dependent effects with increasing divergence between groups over 96 h. **(C)** Colony formation assays with representative images of crystal violet-stained plates and quantitative analysis showing enhanced clonogenic capacity with miR-6756-5p overexpression and reduced colony formation with inhibition (asterisk, p less than 0.05). **(D)** Transwell invasion assays through Matrigel-coated chambers with representative microscopic images and quantitative cell counts demonstrating increased invasive capacity with mimic transfection and decreased invasion with inhibitor treatment (double asterisk, p less than 0.01). **(E)** Cell migration assays using uncoated Transwell chambers with representative images and quantification showing enhanced migratory potential with miR-6756-5p overexpression and reduced migration with knockdown.

To validate the functional significance of hsa-miR-6756-5p, we performed comprehensive *in vitro* experiments using TPC-1 and BHT101 thyroid cancer cell lines. Transfection experiments included mimic (overexpression), inhibitor (knockdown), and negative control conditions. Cell proliferation assays using CCK-8 revealed that hsa-miR-6756-5p overexpression significantly promoted cell growth in both cell lines, while inhibition substantially reduced proliferation rates (Figures 6A,B). In TPC-1 cells, the mimic group showed increased absorbance values compared to negative control at all time points, with the most pronounced difference at 96 h. The inhibitor group showed significantly reduced proliferation compared to control. Similar trends were observed in BHT101 cells, where mimic treatment enhanced proliferation and inhibitor treatment suppressed growth. These effects were time-dependent and consistently observed across multiple time points (24, 48, 72, and 96 h). Colony formation assays further confirmed the growth-promoting effects of hsa-miR-6756-5p, with mimic-transfected cells forming significantly more and larger colonies compared to controls, while inhibitor treatment markedly reduced colony formation capacity (Figure 6C). Quantitative analysis showed that mimic transfection increased colony numbers by approximately 60 percent in TPC-1 cells and 55 percent in BHT101 cells compared to negative controls, while inhibitor treatment reduced colony formation by approximately 70 percent and 65 percent, respectively (p less than 0.05 for all comparisons). The oncogenic properties of hsa-miR-6756-5p extended beyond proliferation, as demonstrated by invasion and migration assays. Transwell invasion assays showed that hsa-miR-6756-5p overexpression significantly enhanced the invasive capacity of both TPC-1 and BHT101 cells, while inhibition substantially reduced invasion through Matrigel-coated chambers (Figure 6D). Microscopic images showed increased numbers of invaded cells in mimic-treated groups and decreased invasion in inhibitor-treated groups. Quantitative analysis revealed that mimic transfection increased invasion by approximately 80 percent in TPC-1 cells and 75 percent in BHT101 cells, while inhibitor treatment reduced invasion by approximately 75 percent and 70 percent, respectively (double asterisk, p less than 0.01 for all comparisons). Similarly, migration assays revealed that hsa-miR-6756-5p positively regulated cell motility, with mimic-transfected cells showing enhanced migratory potential and inhibitor-treated cells displaying reduced migration (Figure 6E). The number of migrated cells was significantly higher in mimic groups and significantly lower in inhibitor groups compared to negative controls in both cell lines.

### Wound healing assays confirm cell motility regulation by hsa-miR-6756-5p

**FIGURE 7:**
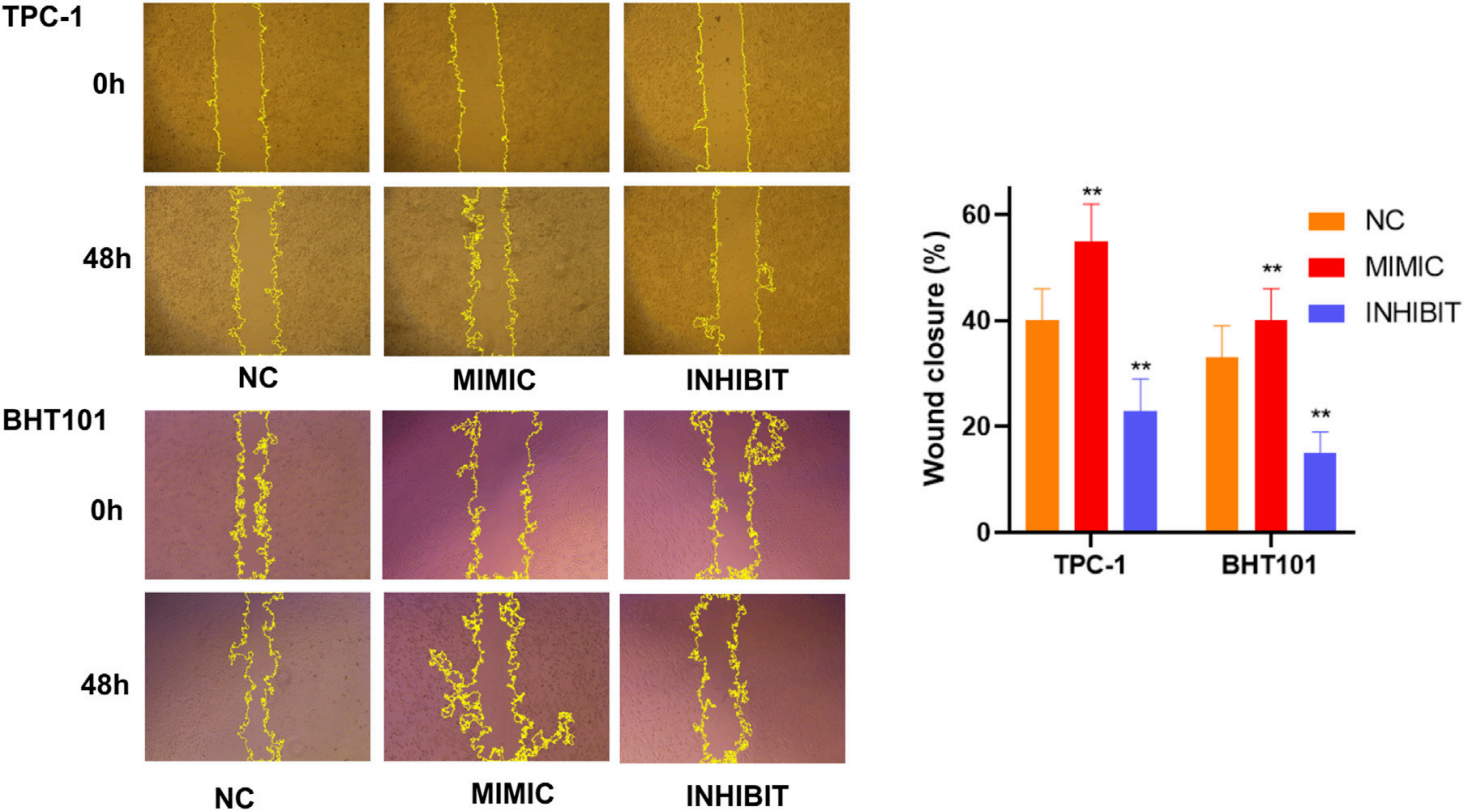
Wound Healing Assays Confirm Cell Motility Regulation by miR-6756-5p. Time-lapse wound healing analysis validates the role of miR-6756-5p in thyroid cancer cell migration. Representative phase-contrast microscopic images of scratch wounds in TPC-1 (upper panels) and BHT101 (lower panels) cells at 0 h and 48 h post-wounding, following transfection with negative control (NC), miR-6756-5p mimic (MIMIC), or inhibitor (INHIBIT). Yellow dashed lines indicate wound boundaries for area measurement. Quantitative analysis reveals significant acceleration of wound closure with miR-6756-5p overexpression: TPC-1 cells achieve approximately 55% closure with mimic versus 40% with control and 22% with inhibitor; BHT101 cells achieve approximately 40% closure with mimic versus 32% with control and 15% with inhibitor. Statistical significance (double asterisk, p less than 0.01) demonstrates consistent effects across both cell lines, reinforcing the critical role of miR-6756-5p in promoting cell migration.

Wound healing scratch assays provided additional evidence for the role of hsa-miR-6756-5p in regulating thyroid cancer cell motility. Time-lapse analysis over 48 h revealed that hsa-miR-6756-5p overexpression significantly accelerated wound closure in both TPC-1 and BHT101 cell lines (Figure 7). Representative phase-contrast microscopic images showed the progression of wound healing at 0 h and 48 h for each treatment group. In TPC-1 cells, mimic-transfected cells achieved approximately 55 percent wound closure compared to 40 percent in negative controls and only 22 percent in inhibitor-treated cells. In BHT101 cells, mimic treatment resulted in approximately 40 percent wound closure compared to 32 percent in controls and 15 percent in inhibitor-treated cells. Quantitative analysis of wound closure percentages showed statistically significant differences between all groups (double asterisk, p less than 0.01). Conversely, hsa-miR-6756-5p inhibition substantially impaired wound healing capacity. These results were consistent across both cell lines, reinforcing the critical role of hsa-miR-6756-5p in promoting thyroid cancer cell migration and potentially metastatic behavior.

### *In Vivo* validation confirms tumor-promoting effects of hsa-miR-6756-5p in xenograft models

**FIGURE 8:**
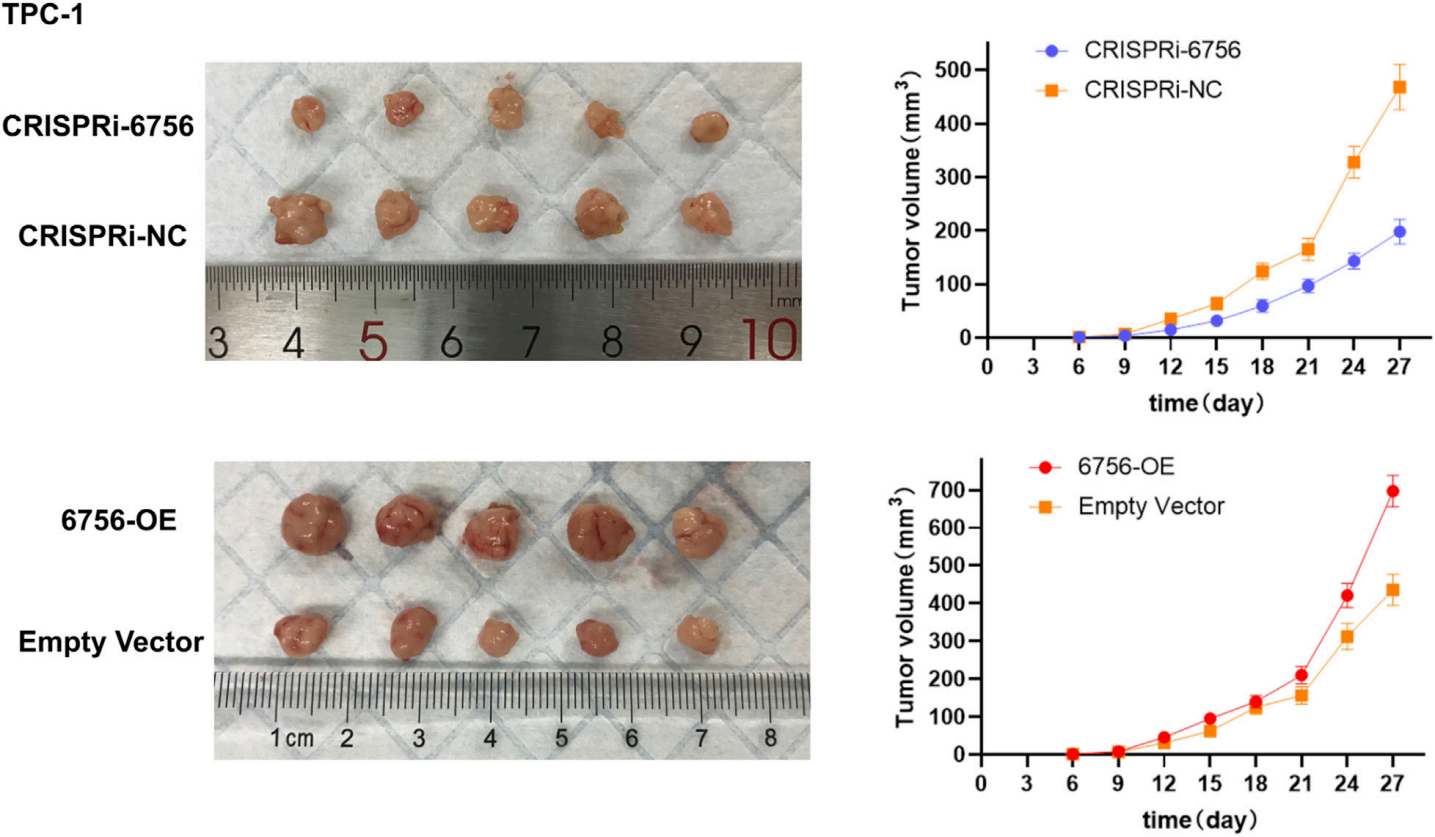
*In Vivo* Validation of miR-6756-5p Tumor-Promoting Effects in Xenograft Models. Subcutaneous xenograft experiments confirm the oncogenic role of miR-6756-5p in thyroid cancer progression. Representative images of excised tumors from nude mice (n equals five per group) injected with TPC-1 cells stably expressing miR-6756-5p (6756-OE), empty vector control, CRISPRi-mediated miR-6756-5p knockdown (CRISPRi-6756), or CRISPRi negative control (CRISPRi-NC), demonstrating visible differences in tumor sizes. Tumor growth curves showing progressive divergence between experimental groups over 27 days miR-6756-5p overexpression significantly enhances tumor development with final volume approximately 700 cubic millimeters versus approximately 450 cubic millimeters in controls (p less than 0.01). Knockdown substantially reduces tumor growth with final volume approximately 200 cubic millimeters versus approximately 470 cubic millimeters in CRISPRi-NC controls (p less than 0.01). Enhanced tumor growth with overexpression becomes evident from day 15 onwards, while growth inhibition with knockdown is apparent throughout the observation period.

To validate our *in vitro* findings in a physiologically relevant context, we conducted xenograft experiments using TPC-1 cells with stable hsa-miR-6756-5p overexpression or CRISPRi-mediated knockdown. Subcutaneous injection of TPC-1 cells into nude mice followed by tumor growth monitoring over 27 days revealed significant differences in tumor development (Figure 8). Representative images of excised tumors from each experimental group demonstrated visible differences in tumor sizes. Mice injected with hsa-miR-6756-5p-overexpressing cells (designated as 6756-OE) developed significantly larger tumors compared to empty vector controls, with final tumor volumes reaching approximately 700 cubic millimeters versus 450 cubic millimeters in controls. The enhanced tumor growth was evident from day 15 onwards, with progressively increasing differences over time. Tumor growth curves showed consistent divergence between groups, with the 6756-OE group demonstrating accelerated growth kinetics. Conversely, mice injected with hsa-miR-6756-5p-knockdown cells (designated as CRISPRi-6756) showed substantially reduced tumor growth, with final volumes of approximately 200 cubic millimeters compared to 470 cubic millimeters in CRISPRi-negative controls. The tumor growth curves demonstrated statistically significant differences between experimental groups and their respective controls at multiple time points (p less than 0.01 for 6756-OE versus control and CRISPRi-6756 versus CRISPRi-NC from day 15 onwards), confirming the tumor-promoting role of hsa-miR-6756-5p *in vivo* and validating its potential as both a diagnostic biomarker and therapeutic target for thyroid cancer.

## Discussion

This study successfully demonstrates the value of integrating bulk transcriptomic analysis with single-cell spatial biomarker mapping and AI-driven analytical approaches for cancer biomarker discovery. Our multi-scale approach, combining meta-analysis of bulk RNA-sequencing data, machine learning-based feature selection, single-cell resolution validation, and comprehensive experimental confirmation, represents a robust framework for identifying clinically relevant biomarkers while ensuring cell-type specificity and tumor microenvironment context. The exceptional diagnostic performance of our four-gene panel in bulk data, achieving AUC values of 0.99–1.0 in training datasets and maintaining good performance (AUC of 0.74) in independent validation, was substantiated by single-cell analysis revealing cell-type-specific expression patterns essential for clinical interpretation.

The discovery that our biomarkers are not exclusively epithelial markers but show multi-cellular expression patterns has important implications for biomarker biology and clinical utility. BID and ITM2A predominantly expressed in epithelial cells likely reflect tumor cell-intrinsic alterations, consistent with their roles in apoptosis regulation and tumor suppression, respectively (Bertran-Alamillo et al., 2023; Jiang et al., 2024). In contrast, TGM2 enrichment in immune and stromal compartments suggests involvement in tumor microenvironment remodeling, extracellular matrix modification, and immune cell function (Chang et al., 2024). This cell-type distribution information, only accessible through single-cell approaches, provides mechanistic insights into biomarker function and supports their utility as comprehensive tumor ecosystem indicators rather than simple tumor cell markers (Zielinski et al., 2021; Hong et al., 2024). The multi-cellular expression pattern suggests that measuring these biomarkers in bulk tissue or liquid biopsies would capture information from multiple cell types, potentially increasing diagnostic sensitivity and providing information about both tumor cells and their microenvironment (Hong et al., 2020a).

The striking finding that immune cells comprise 61.9 percent of analyzed cells in thyroid cancer samples underscores the importance of immune microenvironment in disease pathogenesis and potential therapeutic targeting. This observation aligns with accumulating evidence that the tumor microenvironment plays critical roles in cancer progression, therapeutic response, and clinical outcomes (Hong et al., 2020b; Wang et al., 2023). The differential immune composition between metastatic and non-metastatic samples provides valuable insights into immune landscape changes during disease progression (Yang et al., 2025). The elevated CD8-positive to CD4-positive T cell ratio in distant metastases (1.59) compared to primary tumors (0.87) suggests enhanced cytotoxic immune responses in advanced disease. This finding appears paradoxical given that metastatic disease represents immune escape, but it may reflect several biological scenarios. First, the high CD8-positive T cell infiltration in distant metastases could represent an ongoing but ultimately ineffective immune response, with T cells present but functionally exhausted or inhibited by immunosuppressive mechanisms. Second, the observed pattern might reflect immune selection pressure that shapes tumor evolution, with metastatic clones having acquired immune evasion mechanisms despite high cytotoxic T cell presence. Third, the elevated CD8-positive T cell infiltration could be partially reactive to ongoing tumor cell death and antigen release in rapidly growing metastases.

The dramatically reduced B cell infiltration in distant metastases (1.3 percent) compared to lymph node metastases (22.9 percent) and normal tissues (24.0 percent) provide additional insights into immune microenvironment remodeling. B cells and plasma cells contribute to anti-tumor immunity through antibody production, antigen presentation, and cytokine secretion (Laumont and Nelson, 2023). The progressive loss of B cells during metastatic progression may indicate immune exhaustion, active immune suppression by tumor cells, or alterations in chemokine gradients that normally recruit B cells. This observation suggests that therapeutic strategies aimed at enhancing B cell-mediated immunity might be most effective in early-stage disease or lymph node metastases, where B cell infiltration remains substantial (Laumont et al., 2022). Understanding these immune landscape dynamics has direct implications for immunotherapy approaches and represents the type of spatial biomarker mapping insights that are essential for clinical translation.

The single-cell resolution analysis revealing cell-type-specific expression patterns for our biomarkers provides actionable information for therapeutic development. The predominantly epithelial expression of BID and ITM2A supports their targeting in tumor cells, while TGM2’s enrichment in immune and stromal compartments suggests its modulation could affect tumor-microenvironment interactions. The multi-cellular expression of has-miR-6756-5p observed across epithelial and immune cell types indicates potential for both direct tumor cell targeting and indirect effects through immune modulation. This cell-type specificity information addresses a critical gap regarding the need for empirically grounded, actionable knowledge for clinical translation. Understanding not just which genes are altered, but where in the tumor ecosystem these alterations occur and how they might be therapeutically exploited, is essential for moving biomarkers from discovery to clinical application.

The tumor-specific expression pattern of hsa-miR-6756-5p, being virtually absent in normal tissues but significantly elevated in cancer samples across multiple cell types, makes it an attractive candidate for diagnostic assay development. The single-cell validation confirms this specificity at cellular resolution, supporting development of clinical assays with high specificity and potentially low false-positive rates. The comprehensive functional validation experiments demonstrate that miR-6756-5p functions as a *bona fide* oncogene in thyroid cancer, promoting multiple hallmarks of cancer including enhanced proliferation, colony formation, migration, and invasion. The consistency of these effects across two different thyroid cancer cell lines (TPC-1 and BHT101) strengthens the generalizability of our findings and suggests that miR-6756-5p may play a fundamental role in thyroid cancer pathogenesis regardless of specific genetic backgrounds. The *in vivo* validation using xenograft models further confirms the tumor-promoting effects of miR-6756-5p and supports its potential as a therapeutic target.

However, the specific molecular mechanisms through which miR-6756-5p exerts its oncogenic effects remain to be fully characterized. MicroRNAs typically regulate multiple target genes simultaneously, and identifying the critical targets responsible for the observed phenotypes will require systematic approaches such as AGO2-CLIP sequencing or proteomics. MicroRNAs typically regulate multiple target genes simultaneously, and individual targets may be influenced by multiple microRNAs, creating intricate regulatory networks that require systems-level approaches to fully understand (Hill and Tran, 2021). Future studies should focus on identifying direct target genes and pathways regulated by this microRNA using techniques such as crosslinking immunoprecipitation sequencing (CLIP-seq), RNA sequencing after miR-6756-5p perturbation, and proteomics analysis. Understanding the target gene landscape would reveal the molecular basis of miR-6756-5p′s oncogenic functions and potentially identify additional therapeutic opportunities. The weak correlations observed between miR-6756-3p and the three protein-coding genes (BID, ITM2A, TGM2) suggest complex regulatory relationships that may involve indirect mechanisms or temporal dynamics not captured in our cross-sectional analysis. The lack of significant correlation does not preclude functional relationships, as microRNA-target interactions can be context-dependent and influenced by factors such as competing endogenous RNA networks, RNA-binding proteins, and cellular stress conditions.

Our study demonstrates several methodological advances that could inform future biomarker discovery efforts. The integration of meta-analysis with machine learning approaches provides a framework for identifying robust biomarkers while addressing the reproducibility challenges that have hindered clinical translation of many computational predictions. The use of independent validation datasets and comprehensive experimental validation strengthens confidence in our findings and provides a model for future studies. However, several limitations should be acknowledged. The retrospective nature of our computational analysis means that prospective validation in new patient cohorts will be essential before clinical implementation (Moor et al., 2023). The use of established cell lines, while providing controlled experimental conditions, may not fully recapitulate the complexity and heterogeneity of human thyroid cancer. Additionally, our *in vivo* experiments were conducted in immunocompromised mice, which lack functional adaptive immunity and therefore may not accurately reflect the role of immune responses in thyroid cancer progression in immunocompetent hosts.

The clinical translation potential of our findings is substantial, with several possible applications. The four-gene biomarker panel could be developed into a diagnostic assay for thyroid cancer detection, potentially improving the accuracy of preoperative diagnosis and reducing the need for diagnostic surgery in cases with indeterminate cytology. The tumor-specific expression of miR-6756-5p makes it an attractive target for therapeutic intervention, either through direct targeting with antisense oligonucleotides or locked nucleic acid (LNA) inhibitors, or through modulation of its upstream regulatory mechanisms (Qassem et al., 2025). Furthermore, has-miR-6756-5p could serve as a monitoring biomarker for treatment response or disease recurrence, given its apparent oncogenic functions and specific expression pattern. The development of assays to measure has-miR-6756-5p in liquid biopsies such as blood or fine-needle aspiration samples would be particularly valuable for non-invasive diagnosis and monitoring.

Future research directions should focus on several key areas to advance the clinical application of these findings. First, prospective validation studies in larger patient cohorts are needed to confirm the diagnostic performance of our biomarker panel and establish appropriate cutoff values for clinical use. These studies should include diverse patient populations to ensure generalizability across different ethnic groups, disease stages, and thyroid cancer subtypes. Second, detailed mechanistic studies are required to identify the direct targets and pathways regulated by miR-6756-5p, which could reveal additional therapeutic opportunities and provide insights into thyroid cancer biology. Third, investigation of miR-6756-5p expression and function in other cancer types could determine whether its oncogenic properties are thyroid-specific or represent a broader cancer mechanism (Das et al., 2019). Fourth, development of therapeutic strategies targeting miR-6756-5p, including delivery methods and safety assessments, will be crucial for clinical translation. Finally, integration of our biomarkers with existing clinical and pathological parameters in multivariate models could enhance risk stratification and treatment selection.

In conclusion, our study successfully demonstrates that integration of bulk transcriptomics, single-cell RNA-sequencing, and AI-driven analytical approaches provides comprehensive biomarker validation spanning molecular discovery to cellular contextualization. Machine learning-based forward feature selection was employed to identify the optimal combination of biomarkers from differentially expressed genes. This approach offers advantages over traditional statistical methods by systematically evaluating multiple gene combinations and identifying panels with superior diagnostic performance while minimizing overfitting through cross-validation. The identification of a four-gene diagnostic panel with exceptional performance, validated at single-cell resolution revealing cell-type-specific expression patterns and immune microenvironment associations, exemplifies multi-scale biomarker characterization. The tumor-specific expression and oncogenic functions of miR-6756-5p confirmed through extensive experimental validation provide both a diagnostic biomarker and therapeutic target for thyroid cancer. Our findings support continued integration of computational prediction, single-cell spatial mapping, and experimental validation as the optimal framework for developing clinically translatable cancer biomarkers.

## Data Availability

The datasets presented in this study can be found in online repositories. The names of the repository/repositories and accession number(s) can be found in the article/supplementary material.

## References

[B1] Affinito O. Orlandella F. M. Luciano N. Salvatore M. Salvatore G. Franzese M. (2022). Evolution of intra-tumoral heterogeneity across different pathological stages in papillary thyroid carcinoma. Cancer cell Int. 22, 263. 10.1186/s12935-022-02680-1 35996174 PMC9394008

[B2] Aswathy R. Chalos V. A. Suganya K. Sumathi S. (2024). Advancing miRNA cancer research through artificial intelligence: from biomarker discovery to therapeutic targeting. Med. Oncol. N. Lond. Engl. 42, 30. 10.1007/s12032-024-02579-z 39688780

[B3] Bertran-Alamillo J. Giménez-Capitán A. Román R. Talbot S. Whiteley R. Floc'h N. (2023). BID expression determines the apoptotic fate of cancer cells after abrogation of the spindle assembly checkpoint by AURKB or TTK inhibitors. Mol. cancer 22, 110. 10.1186/s12943-023-01815-w 37443114 PMC10339641

[B4] Borzooei S. Briganti G. Golparian M. Lechien J. R. Tarokhian A. (2024). Machine learning for risk stratification of thyroid cancer patients: a 15-year cohort study. Eur. archives oto-rhino-laryngology 281, 2095–2104. 10.1007/s00405-023-08299-w 37902840

[B5] Cao S. Yin Y. Hu H. Hong S. He W. Lv W. (2023). “CircGLIS3 inhibits thyroid cancer invasion and metastasis through miR-146b-3p/AIF1L axis,” Cell. Oncol. 46, 1777–1789. 10.1007/s13402-023-00845-2 37610691 PMC12974756

[B6] Chang W. Gao W. Liu D. Luo B. Li H. Zhong L. (2024). The upregulation of TGM2 is associated with poor prognosis and the shaping of the inflammatory tumor microenvironment in lung squamous cell carcinoma. Am. J. cancer Res. 14, 2823–2838. 10.62347/OBES4130 39005693 PMC11236791

[B7] Chen D. W. Lang B. H. H. McLeod D. S. A. Newbold K. Haymart M. R. (2023). Thyroid cancer. Thyroid. cancer. Lancet London, Engl. 401, 1531–1544. 10.1016/S0140-6736(23)00020-X 37023783

[B8] Chorley B. N. Atabakhsh E. Doran G. Gautier J. C. Ellinger-Ziegelbauer H. Jackson D. (2021). Methodological considerations for measuring biofluid-based microRNA biomarkers. Crit. Rev. Toxicol. 51, 264–282. 10.1080/10408444.2021.1907530 34038674 PMC8577439

[B9] Das D. K. Persaud L. Sauane M. (2019). MicroRNA-4719 and microRNA-6756-5p correlate with castration-resistant prostate cancer progression through Interleukin-24 regulation. Non-coding RNA 5, 10. 10.3390/ncrna5010010 30669553 PMC6468726

[B10] Dlamini Z. Francies F. Z. Hull R. Marima R. (2020). Artificial intelligence (AI) and big data in cancer and precision oncology. Comput. Struct. Biotechnol. J. 18, 2300–2311. 10.1016/j.csbj.2020.08.019 32994889 PMC7490765

[B11] Hill M. Tran N. (2021). “miRNA interplay: mechanisms and consequences in cancer,” in Disease models and mechanisms 14. 10.1242/dmm.047662 PMC8077553 33973623

[B12] Hong W. Yuan H. Gu Y. Liu M. Ji Y. Huang Z. (2020a). Immune-related prognosis biomarkers associated with osteosarcoma microenvironment. Cancer cell Int. 20, 83. 10.1186/s12935-020-1165-7 32190007 PMC7075043

[B13] Hong W. Gu Y. Guan R. Xie D. Zhou H. Yu M. (2020b). Pan-cancer analysis of the CASP gene family in relation to survival, tumor-infiltrating immune cells and therapeutic targets. Genomics 112, 4304–4315. 10.1016/j.ygeno.2020.07.026 32682809

[B14] Hong W. Zhang Y. Wang S. Zheng D. Hsu S. Zhou J. (2024). Deciphering the immune modulation through deep transcriptomic profiling and therapeutic implications of DNA damage repair pattern in hepatocellular carcinoma. Cancer Lett. 582, 216594. 10.1016/j.canlet.2023.216594 38135208

[B15] Jiang J. Xu J. Ou L. Yin C. Wang Y. Shi B. (2024). ITM2A inhibits the progression of bladder cancer by downregulating the phosphorylation of STAT3. Am. J. cancer Res. 14, 2202–2215. 10.62347/KHCC9690 38859860 PMC11162684

[B16] Laumont C. M. Nelson B. H. (2023). B cells in the tumor microenvironment: Multi-faceted organizers, regulators, and effectors of anti-tumor immunity. Cancer cell 41, 466–489. 10.1016/j.ccell.2023.02.017 36917951

[B17] Laumont C. M. Banville A. C. Gilardi M. Hollern D. P. Nelson B. H. (2022). Tumour-infiltrating B cells: immunological mechanisms, clinical impact and therapeutic opportunities. Nat. Rev. Cancer 22, 414–430. 10.1038/s41568-022-00466-1 35393541 PMC9678336

[B18] Li W. Liu Z. Cen X. Xu J. Zhao S. Wang B. (2022). Integrated analysis of fibroblasts molecular features in papillary thyroid cancer combining single-cell and bulk RNA sequencing technology. Front. Endocrinol. 13, 1019072. 10.3389/fendo.2022.1019072 36387901 PMC9643292

[B19] Ligero M. El Nahhas O. S. M. Aldea M. Kather J. N. (2025). Artificial intelligence-based biomarkers for treatment decisions in oncology. Trends cancer 11, 232–244. 10.1016/j.trecan.2024.12.001 39814650

[B20] Lin S. Zhu Y. Ji C. Yu W. Zhang C. Tan L. (2022). METTL3-Induced miR-222-3p upregulation inhibits STK4 and promotes the malignant behaviors of thyroid carcinoma cells. J. Clin. Endocrinol. metabolism 107, 474–490. 10.1210/clinem/dgab480 34562008

[B21] Liu J. B. Ramonell K. M. Carty S. E. McCoy K. L. Schaitkin B. M. Karslioglu-French E. (2023). Association of comprehensive thyroid cancer molecular profiling with tumor phenotype and cancer-specific outcomes. Surgery 173, 252–259. 10.1016/j.surg.2022.05.048 36272768 PMC11189592

[B22] Maleki F. Ovens K. Gupta R. Reinhold C. Spatz A. Forghani R. (2023). Generalizability of machine learning models: quantitative evaluation of three methodological pitfalls. Artif. Intell. 5, e220028. 10.1148/ryai.220028 36721408 PMC9885377

[B23] Mann M. Kumar C. Zeng W. F. Strauss M. T. (2021). Artificial intelligence for proteomics and biomarker discovery. Cell Syst. 12, 759–770. 10.1016/j.cels.2021.06.006 34411543

[B24] Moleti M. Aversa T. Crisafulli S. Trifirò G. Corica D. Pepe G. (2023). Global incidence and prevalence of differentiated thyroid cancer in childhood: systematic review and meta-analysis. Front. Endocrinol. 14, 1270518. 10.3389/fendo.2023.1270518 37795368 PMC10546309

[B25] Moor M. Bennett N. Plečko D. Horn M. Rieck B. Meinshausen N. (2023). Predicting sepsis using deep learning across international sites: a retrospective development and validation study. EClinicalMedicine 62, 102124. 10.1016/j.eclinm.2023.102124 37588623 PMC10425671

[B26] Moradi Kashkooli F. Hornsby T. K. Kolios M. C. Tavakkoli J. J. (2024). Ultrasound-mediated nano-sized drug delivery systems for cancer treatment: Multi-scale and multi-physics computational modeling. Wiley Interdiscip. Rev. Nanomedicine nanobiotechnology 16, e1913. 10.1002/wnan.1913 37475577

[B27] Nabhan F. Dedhia P. H. Ringel M. D. (2021). Thyroid cancer, recent advances in diagnosis and therapy. Int. J. cancer 149, 984–992. 10.1002/ijc.33690 34013533

[B28] Oskouie A. A. Ahmadi M. S. Taherkhani A. (2022). Identification of prognostic biomarkers in papillary thyroid cancer and developing non-invasive diagnostic models through integrated bioinformatics analysis. MicroRNA Shariqah, United Arab. Emir. 11, 73–87. 10.2174/2211536611666220124115445 35068400

[B29] Patel A. U. Mohanty S. K. Parwani A. V. (2022). Applications of digital and computational pathology and artificial intelligence in genitourinary pathology diagnostics. Surg. Pathol. Clin. 15, 759–785. 10.1016/j.path.2022.08.001 36344188

[B30] Qassem S. Naidu G. S. Goldsmith M. Breier D. Rampado R. Ramishetti S. (2025). Targeting intestinal inflammation using locked nucleic acids delivered *via* lipid nanoparticles. Nat. Commun. 16, 7682. 10.1038/s41467-025-63037-6 40825982 PMC12361575

[B31] Ruiz-Pozo V. A. Cadena-Ullauri S. Guevara-Ramírez P. Paz-Cruz E. Tamayo-Trujillo R. Zambrano A. K. (2023). Differential microRNA expression for diagnosis and prognosis of papillary thyroid cancer. Front. Med. 10, 1139362. 10.3389/fmed.2023.1139362 37089590 PMC10113479

[B32] Schlumberger M. Leboulleux S. (2021). Current practice in patients with differentiated thyroid cancer. Nat. Rev. Endocrinol. 17, 176–188. 10.1038/s41574-020-00448-z 33339988

[B33] Sun T. Guan Q. Wang Y. Qian K. Sun W. Ji Q. (2021). Identification of differentially expressed genes and signaling pathways in papillary thyroid cancer: a study based on integrated microarray and bioinformatics analysis. Gland. Surg. 10, 629–644. 10.21037/gs-20-673 33708546 PMC7944059

[B34] Sun C. X. Liu B. J. Su Y. Shi G. W. Wang Y. Chi J. F. (2022). MiR-181a promotes cell proliferation and migration through targeting KLF15 in papillary thyroid cancer. Clin. and Transl. Oncol. 24, 66–75. 10.1007/s12094-021-02670-1 34312797

[B35] Tan J. K. Awuah W. A. Roy S. Ferreira T. Ahluwalia A. Guggilapu S. (2023). Exploring the advances of single-cell RNA sequencing in thyroid cancer: a narrative review. Med. Oncol. N. Lond. Engl. 41, 27. 10.1007/s12032-023-02260-x 38129369 PMC10739406

[B36] Wang J. Lv N. Lu X. Yuan R. Chen Z. Yu J. (2021). Diagnostic and therapeutic role of microRNAs in oral cancer. Oncol. Rep. 45, 58–64. 10.3892/or.2020.7854 33200230 PMC7709826

[B37] Wang Q. Shao X. Zhang Y. Zhu M. Wang F. X. C. Mu J. (2023). Role of tumor microenvironment in cancer progression and therapeutic strategy. Cancer Med. 12, 11149–11165. 10.1002/cam4.5698 36807772 PMC10242329

[B38] Wang N. Hong W. Wu Y. Chen Z. S. Bai M. Wang W. (2024). Next-generation spatial transcriptomics: unleashing the power to gear up translational oncology. MedComm 5, e765. 10.1002/mco2.765 39376738 PMC11456678

[B39] Yang F. Zhang J. Li B. Zhao Z. Liu Y. Zhao Z. (2021). Identification of potential lncRNAs and miRNAs as diagnostic biomarkers for Papillary thyroid carcinoma based on machine learning. Int. J. Endocrinol. 2021, 3984463. 10.1155/2021/3984463 34335744 PMC8318749

[B40] Yang H. Liu D. Qiu L. Wang R. Zhang C. Yu D. (2025). Reprogramming cellular senescence and aging clocks for advanced cancer immunotherapy. Mol. cancer 24, 237. 10.1186/s12943-025-02459-8 41039629 PMC12492661

[B41] Zhu Z. C. Li T. Yao T. Q. Bi J. C. Jiao L. H. (2022). Changes of serum miR-221 and miR-145 levels with papillary thyroid carcinoma and their relationship with invasive activity. Cell. Mol. Biol. (Noisy-le-Grand, France) 67, 293–301. 10.14715/cmb/2021.67.5.40 35818240

[B42] Zielinski J. M. Luke J. J. Guglietta S. Krieg C. (2021). High throughput multi-omics approaches for clinical trial evaluation and drug discovery. Front. Immunol. 12, 590742. 10.3389/fimmu.2021.590742 33868223 PMC8044891

